# SOCIAL INTELLIGENCE TRAINING FOR CUSTODIAL GRANDMOTHER-ADOLESCENT GRANDCHILDREN DYADS

**DOI:** 10.1093/geroni/igz038.1295

**Published:** 2019-11-08

**Authors:** Britney A Webster, Greg Smith, Frank Infurna

**Affiliations:** 1 Kent State University, Kent, Ohio, United States; 2 Arizona State University, Tempe, Arizona, United States

## Abstract

Custodial grandmothers (CGMs) and adolescent custodial grandchildren (ACG) face risk of poorer social skills and competencies due to early life adversities which have downstream negative consequences for mental and physical health. We describe an RCT examining the efficacy of an online social intelligence intervention (SII) at improving the emotional, interpersonal, and physical well-being of CGM-ACG dyads through mutual enhancement of their social competencies. Our SII is particularly valuable for these dyads because it enhances their social competencies and relationships, thereby leading to positive outcomes. Additionally, adolescence is a critical period for developing social competencies, largely through interactions with female caregivers. Our longitudinal mixed-methods approach addresses four aims: (1) Investigating if SII improves social competencies and overall well-being through both actor and partner effects; (2) Exploring moderators of SII efficacy; (3) Studying qualitatively how dyads view SII as changing their lives; and (4) Conducting a SII cost-benefit analysis. [Funded by R01AG054571]

